# How Does Smart Healthcare Service Affect Resident Health in the Digital Age? Empirical Evidence From 105 Cities of China

**DOI:** 10.3389/fpubh.2021.833687

**Published:** 2022-01-21

**Authors:** Yan Chen, Liyezi Zhang, Mengyang Wei

**Affiliations:** School of Economics and Management, Beijing University of Posts and Telecommunications, Beijing, China

**Keywords:** smart healthcare service, resident health, utilization of healthcare service, outpatient services, inpatient services, healthcare service innovation, health behaviors

## Abstract

With the emergence of the digital age, smart healthcare services based on the new generation of information technologies play an increasingly important role in improving the quality of resident health. This study empirically examined the impact of regional smart healthcare services on resident health as well as the underlying mechanism by employing a two-way fixed effects model. We constructed a Regional Smart Healthcare Service Development Index and matched it with survey data from the China Health and Retirement Longitudinal Study to validate the model. The results showed that (1) smart healthcare services have a significant positive impact on resident health. (2) The availability of outpatient services and inpatient services plays a mediating role in the relationship between regional smart healthcare services and resident health. (3) The influence of regional smart healthcare services on resident health is heterogeneous among different regions. Specifically, the effect of smart healthcare services on resident health is significant in the eastern regions, while it is not significant in the central, western, and northeastern regions. The effect of smart healthcare services on resident health is significant in rural regions but not in urban regions. This study enriches the nascent research stream of smart healthcare services. This study offers useful insights for practitioners and the government to guide them in formulating smart healthcare strategies.

## Introduction

In recent years, there has been a growing academic interest in healthcare service innovation (1). Superior healthcare services can provide a solid foundation for national competitiveness and societal soundness (2). However, the current traditional healthcare service system is inherently affected by geographical limits and uneven distribution of healthcare resources, which are relatively powerless in responding to unusual events, such as unpredictable public health crises (3). In particular, the outbreak of COVID-19 has shown a pressing need for global healthcare industries to enhance their flexibility and responsiveness by innovating service models (4).

Among the innovative models, the smart healthcare service is the most promising approach, attracting a great deal of attention from both academics and practitioners. Smart healthcare services refer to embedding the new generation of intelligent technologies into the traditional healthcare industry to provide patients with more convenient, flexible, personalized, and diversified healthcare services (5, 6). The application of intelligent technologies has changed the way of value creation of service systems by improving medical devices, optimizing treatment processes, and empowering disease diagnosis and prediction, which leads to the transformation of the organizational structure, ecosystem, and innovative model of the modern healthcare service industry (7–9).

Scholars have attempted to understand how to incorporate various intelligent technologies into the healthcare service industry. Sensor network technology and wearable devices can collect patient data continuously, enabling real-time monitoring of patient health status (10). The machine collaboration clinical intelligent diagnosis systems and surgical robots within artificial intelligence have been applied in pathological classification and intelligent consultation, playing an increasingly important role in epidemiological surveillance and prevention (11). Blockchain technology opens a new possibility for the healthcare service sector for data sharing, health record management, and access control (12). Owing to its durability, transparency, immutability, and process integrity, blockchain can effectively address the problem of weak privacy protection and inefficient data management (13). Moreover, the progress of 5G and the Internet of Things (IoT) provides strong technical support for breaking geographical restrictions to provide innovative telemedicine services (14). Overall, the present literature has presented that information and communication technology tools have huge potential to help the healthcare sector overcome shortages and spawn new innovative service models.

In addition to the feasibility of various intelligent technologies in the healthcare industry, existing studies have also analyzed the multiple advantages of smart healthcare services. First, the smart healthcare service overcomes the shortcomings of the low efficiency of traditional outpatient services by allowing doctors to make the diagnosis in an appropriate time according to the urgency of patients (15). Second, compared with the traditional model, telemedicine service avoids the risk of cross-infection resulting from face-to-face contact, which is a cost-effective strategy for preliminary evaluation of acute patients (16). Third, under the smart healthcare service model, healthcare functions, patients, and doctors are connected in an intelligent and scalable health ecosystem, which is conducive to realizing the cross-departmental diagnosis and improving the accuracy of prediction and diagnosis (17).

Previous studies have explored the motivations, drivers, barriers, and innovation of smart healthcare service adoption in public health from the micro-organizational perspective (5, 6, 18, 19). Nevertheless, the analysis of the impact of smart healthcare services from a meso-industrial or macro-regional perspective remains unclear. While scholars have claimed that the ultimate goal of innovating healthcare services is to enhance resident health, surprisingly, little empirical evidence regarding the effect of innovative smart healthcare services on resident health can be found in the literature. In addition, the mechanisms through which smart healthcare services enable resident health remain unclear. Accordingly, our study proposes the following two research questions (RQs):

RQ1: What is the impact of smart healthcare services on resident health?

RQ2: Through what mechanisms do smart healthcare service influence resident health?

To answer these questions, we developed a theoretical model and validated it using data from the China Health and Retirement Longitudinal Study (CHARLS), which was carried on the Institute of Social Science Survey, Peking University. CHARLS data are widely used in studies of healthcare and population aging (20, 21). This study makes three theoretical contributions to the literature. Firstly, the paper fills a research gap related to the impact of smart healthcare services on resident health at the regional level, which enriches the research perspective of smart healthcare services. Secondly, the paper attempts to clarify the mechanism by which smart healthcare services affect resident health by incorporating the two mediating factors from the perspective of healthcare service availability, which fills the theoretical gaps in regional performance research on smart healthcare services. Thirdly, the paper develops the study on dwindling the differences in various regions through smart healthcare services. Concerning practical implications, this study offers useful insights into the construction of smart healthcare service infrastructure and the implementation of dynamic and differentiated regional strategies by the government.

The remainder of this study is organized as follows. Section Theoretical Underpinning and Hypotheses Development provides theoretical underpinning and develops the hypotheses. In section Data Sources and Specification of Variables, we describe our research methodology, including construct measurements, sample selection, and data collection. Section Model Selection and Empirical Result presents the data analysis and the results. Finally, we summarize the research conclusions, theoretical contributions, and provide practical suggestions in Section Further Discussion.

## Theoretical Underpinning and Hypotheses Development

In general, smart healthcare services can effectively improve resident health by increasing the efficiency and quality of healthcare services. The enhanced efficiency of smart healthcare services is mainly reflected in the following three aspects. First, smart healthcare services can break geographical restrictions and simplify disease diagnosis process. Specifically, telemedicine service overcomes geographical barriers, which provides great convenience for patients with limited mobility or underdeveloped areas (22). At the same amount of time, telemedicine system processed 580 requests of patients while human processed only 256 patients (23). Hence, it can be seen that smart healthcare services have superiority in simplifying disease diagnosis process and reducing patients' waiting time for services. Second, smart healthcare services provide more flexible service models to realize an immediate response to the service requirements. Characterized by service-oriented network architecture, 5G communication technology can flexibly expand the communication protocol interfaces and network functions according to service requirements (24, 25). Finally, smart healthcare services play a crucial role in assisting doctors in quickly judging the condition. Using big data mining to analyze the information of patients, doctors are enabled to judge patients' conditions quickly and make timely diagnosis decisions (26).

With respect to the quality of healthcare services, smart healthcare services can play a role in several ways. First, the cost of smart healthcare services is reduced owing to network externalities. Smart healthcare services are based on network infrastructure, thereby having the characteristics of network externalities. Network externality means that the value of connecting to a network depends on the number of people already connected to the network (27). Simultaneously, according to Metcalfe's Law, the network value is proportional to the square of its users. Namely, the more users flow in the network, the stronger the network scale effect. When the total number of users reaches a critical value, the “winner-take-all” phenomenon appears (28). Therefore, smart healthcare services enable residents to obtain high-quality healthcare services at a relatively lower cost. Second, smart healthcare services construct a value co-creation model involving multiple participants, which contributes to improve service quality. Doctors, patients, and other participants realize interconnection through the smart healthcare service platform system, promoting the flow of elements such as medical data, knowledge, and technology. Moreover, according to the resource dependence theory, healthcare organizations could form interdependency relationships through cooperative alliances (29). The employment of information technology can strengthen such alliance relationship by deepening interconnection of healthcare organizations among regions. A smart healthcare service system can facilitate the information sharing, resource integration, and value co-creation across healthcare organizations among different regions (30, 31). Finally, smart healthcare services realize a transition from a clinic-centric-centric treatment to patient-centric healthcare models (32). It is instrumental in healthcare institutions to transform mental healthcare into more personalized medical service solutions to purposefully solve patients' health problems (33). Based on the above analysis, we propose H1:

H1: Smart healthcare service is positively correlated with resident health.

In addition to the direct effect discussed above, smart healthcare services have an indirect effect on resident health by enhancing the supply capacity of healthcare services. Based on the types of healthcare service mode, the supply capacity of healthcare services can be roughly measured by the availability of outpatient and inpatient services (34). Regarding outpatient services, smart medical services can decrease the use of offline outpatient services, thus improving resident health (35). First, telemedicine services greatly improve the immediacy, convenience, and diversity of communication methods between doctors and patients (36). Second, smart healthcare services play an important role in diagnosing infectious diseases and reducing the risk of cross-contamination caused by face-to-face contact. For example, during COVID-19, patients have showed a higher willingness to use video consultations than before (37). Based on telemedicine service systems and intelligent medical robots, patients can be classified, evaluated, monitored, and treated within a safe distance, which reduces the risk of frontline medical staff infected with COVID-19. Third, smart healthcare service systems apply blockchain technology to information-intensive electronic healthcare records, which contributes to privacy protection of patients and reducing the risk of misusing health information in a very short time (38). Following the above analysis, we propose H2a:

H2a: Smart healthcare services will improve resident health by decreasing the use of outpatient services.

Concerning inpatient services, smart healthcare services can enhance the availability of inpatient services, thus improving resident health. Demand for inpatient services is limited by the infrastructure of healthcare institutions to an extent, especially the number of available hospital beds and medical devices. The unreasonable distribution of resources is an important problem hindering the development of China's hospitals and the medical system. On the one hand, smart healthcare services could optimize allocation of resources among different medical institutions, thereby improving the availability of inpatient services and meeting the healthcare needs of patients (39). In China, most medical resources are concentrated in large hospitals owing to urbanization (40). As the expansion of hospitals cannot keep up with the growth of patients, large medical institutions face the dilemma of excessive pressure and low availability of medical resources. The lack of a perfect referral system and patients' prejudices against general practice in China have led patients to tend to large medical institutions, even for the common cold (41). The smart healthcare service system can realize real-time information sharing in different medical institutions among regions, which is helpful in improving the inpatient service capacity of small- and medium-sized medical institutions and release the pressure of large medical institutions. On the other hand, smart healthcare services play an important role in healthcare crisis management. When public health emergencies occur, many countries face the problem of a shortage of intensive care resources. The most recent COVID-19 outbreak is a typical example. During COVID-19, the demand for intensive care resources, especially for intensive care unit beds and ventilators, rose sharply in China. Hence, effectively allocating resources become increasingly important. The smart healthcare system assists hospitals and medical facilities in making quicker decisions that have a higher priority to be hospitalized, which makes for the effective allocation of scarce medical resources (42). Concurrently, the smart healthcare system automatically conducts triage to reduce deaths caused by necessary care delay (43). Based on the above analysis, we propose H2b:

H2b: Smart healthcare services improve resident health by increasing the availability of inpatient services.

## Data Sources and Specification of Variables

### Data Sources

The study data included two parts. Of these, the data of dependent variables and mediating variables were derived from the survey mentioned above—the CHARLS. The data for the independent variable were derived from the multi-index system we constructed in this study—the Regional Smart Healthcare Service Development Index. Specifically, CHARLS is a national tracking survey that collects data representing families and individuals of residents aged 45 years or older in China. The multi-stage stratified sampling method is mainly used to conduct a field survey in 140 counties from 30 provinces in China; the baseline survey started in 2011, and a follow-up survey was conducted every 2–3 years. We selected survey data from 2011, 2013, 2015, and 2018 as the samples for this study.

Previous studies have generally explored the application of smart healthcare services in the context of smart cities (44, 45). Smart city services, smart applications, and smart devices are the basic elements of smart healthcare services (46). Considering the basic elements of smart healthcare services, we constructed a comprehensive development index—the Regional Smart Healthcare Service Development Index—by integrating data from three dimensions: regional, industrial, and organizational. The regional data were collected from the China Urban Statistical Yearbook and Chinese Research Data Services Platform database. The healthcare industry data were obtained from the China Health Statistical Yearbook, and smart healthcare service enterprise data were drawn from the Qixinbao database.

We obtained the sample data of this study by matching the two types of data according to the location of the respondents. Excluding the 7,173 respondents' missing key information, the final sample data contain 35,124 samples of 8,781 respondents from 105 cities in China.

### Variables Selection

#### Dependent Variables

The dependent variable was resident health, measured by self-rated health. Self-rated health indicates the objective and subjective perception of one's health and is one of the most frequently employed health indicators in sociological health research (47, 48). Respondents were asked to respond to the question, “Would you say your health is very good, good, fair, poor or very poor?” using a five-point Likert scale. The higher the score, the better the health.

#### Independent Variables

The independent variable was the Regional Smart Healthcare Service Development Index. Considering the characteristics of smart healthcare services and the availability of data, we measured it from two aspects: smart medical infrastructure and smart service foundation (49, 50). The smart medical infrastructure includes the number of regional medical institutions, the number of beds, and doctors in medical institutions. The smart service foundation includes the number of smart healthcare equipment manufacturing companies, the number of regional telecom services, and the number of regional Internet users. In particular, we obtained the number of smart healthcare equipment manufacturing companies by screening the industry codes of smart healthcare services. The industry codes are determined based on the Statistical Classification of Digital Economy and Core Industries (2021) published by the China National Bureau of Statistics in May 2021. Qixinbao is a database that uses the Chinese enterprise credit information publicity system as the data source. We searched for the companies with the above industry codes in the Qixinbao database and obtained the specific number of smart healthcare equipment manufacturing companies in each city. The specific indicators of the smart healthcare service development index are shown in Table 1. Through the principal component analysis method to standardize and reduce the dimensions of the data, we obtained the Regional Smart Healthcare Service Development Index.

**Table 1:** Regional smart healthcare service development index.

**Name**	**Index**	**Meaning**
Regional smart healthcare service development index	Smart medical infrastructure	The number of regional medical institutions
		The number of beds in medical institutions
		The number of doctors in medical institutions
	Smart service foundation	The number of smart healthcare equipment manufacturing companies
		The number of telecom services
		The number of Internet users

#### Mediating Variable

The mediating variables in this study were the availability of outpatient and inpatient services. Specifically, we used the answer to the question, “In the last month, have you visited a public hospital, private hospital, public health center, clinic, or health worker's or doctor's practice, or been visited by a health worker or doctor for outpatient care?” as the proxy variable for the availability of outpatient services and the answer to question “Have you received inpatient care in the past year?” as a proxy variable for the availability of inpatient services.

#### Control Variables

The control variables included the individual control variables and regional control variables. Individual control variables were age, sex, household registration, education level, marital status, chronic diseases, and health insurance of respondents. Regional control variables included regional gross domestic product (GDP) per capita and the level of urbanization. The specific definitions and assignment of the variables are shown in Table 2.

**Table 2:** Variable definition and assignment.

**Variable type**	**Variable code**	**Variable assignment**
Dependent variables	Health	From 1 to 5, the better the health, the higher the value.
Independent variables	SS	Development level of regional smart healthcare services
Mediating variable	OS	Yes = 1, No = 0
	IS	Yes = 1, No = 0
Control variables	Age	Age of respondents
	Sex	Male = 1, Female = 0
	Household	Agricultural household registration = 1, Non-agricultural household registration = 0
	Edu	From 1 to 11, the higher the level of education, the higher the value.
	Mar	Married and living with their spouse=1, other=0.
	Chronic	From 0 to 14, the number of chronic diseases the respondents had.
	Insurance	Yes = 1, No = 0
	GDPP	Reginal GDP per capita
	Urban	Population density (person/sq.km)

### Descriptive Statistics

Descriptive statistics of the variables are presented in Table 3. The D-value of the maximum value and the minimum value of smart healthcare services was ~12, indicating that there were significant differences in the development of smart healthcare services in different regions. The average self-tested health was 3.041, which indicates that resident health status was fair and required improvement. The average outpatient utilization rate and inpatient utilization rate were 0.194 and 0.122, respectively, meaning that the proportion of outpatient services at least once a month and the proportion of inpatient services in the past year were relatively low.

**Table 3:** Descriptive statistics of variables.

**Variable**	**Obs**	**Mean**	**Std. Dev**.	**Min**	**Max**
Health	35124	3.041	0.949	1	5
SS	35124	−7.72E-10	1.345	−1.328	10.636
OS	35124	0.194	0.395	0	1
IS	35124	0.122	0.328	0	1
Age	35124	60.878	8.854	43	108
Sex	35124	0.475	0.499	0	1
Household	35124	0.811	0.392	0	1
Edu	35124	3.434	1.901	1	10
Mar	35124	0.833	0.373	0	1
Chronic	35124	0.621	1.026	0	10
Insurance	35124	0.964	0.186	0	1
GDPP	35124	87094.951	385312	8877	5955349
Urban	35124	570.635	779.086	9.787	11559.601

## Model Selection and Empirical Result

### Model Selection

We developed the following two-way fixed effects model to test the impact of smart healthcare services on the resident health of H1.


(1) $$Health_{it}=\beta_{0}+\beta_{1}SS_{it}+\beta_{2}X_{it}+\mu_{i}+\delta_{t}+\varepsilon_{it}$$

To test the mediating effect of H2a and H2b, we constructed the following mediating effect model by adding the mediating variables, outpatient service availability (OS), and inpatient service availability (IS).


(2) $$OS_{it}=\beta_{0}+\beta_{1}SS_{it}+\beta_{2}X_{it}+\mu_{i}+\delta_{t}+\varepsilon_{it}$$

(3) $$IS_{it}=\beta_{0}+\beta_{1}SS_{it}+\beta_{2}X_{it}+\mu_{i}+\delta_{t}+\varepsilon_{it}$$

(4) $$\begin{array}{l} Health_{it}=\beta_{0}+\beta_{1}SS_{it}+\beta_{2}OS_{it}+\beta_{3}IS_{it}+\beta_{4}X_{it}+\mu_{i}+\delta_{t}\\ \;+\varepsilon_{it} \end{array}$$

where i represents the city (*I* = 1,2…105) and t represents the year (*t* = 2011,2013,2015,2018), μ_i_ indicates city-fixed effects, and δ_t_ indicates year-fixed effects. The dependent variable Health_it_ indicates the health status of residents, and the independent variable SS_it_ represents the development level of the regional smart healthcare service. X_it_ represents control variables affecting residents' health, and ε_it_ represents the random perturbation term.

### Empirical Result

The results of the benchmark regression and mediating effects are shown in Table 4. In Models (1) and (2), the coefficients of smart healthcare service (SS) are all significantly positive (*p* < 0.01), indicating that the development of smart healthcare services can effectively improve resident health. Thus, H1a is supported. Concerning the control variables in Model (2), the results indicate that higher education, health insurance, and urbanization level are helpful in improving resident health, while chronic diseases and GDP per capita were harmful to resident health. Compared with women and rural residents, men and urban residents have advantages in the impact of smart healthcare services on health.

**Table 4:** The results of benchmark regression and mediating effects.

**Variables**	**(1)**	**(2)**	**(3)**	**(4)**	**(5)**	**(6)**
	**Health**	**Health**	**OS**	**IS**	**Health**	**Health**
SS	0.031^***^	0.015^***^	−0.023^***^	0.01^**^	0.014^***^	0.014^***^
	(0.004)	(0.004)	(0.007)	(0.001)	(0.004)	(0.004)
Age		−0.007^***^	−0.001	0.003^***^	−0.007^***^	−0.006^***^
		(0.001)	(0.001)	(0.001)	(0.001)	(0.001)
Sex		0.067^***^	0.096^*^	−0.005	0.061^***^	0.066^***^
		(0.011)	(0.053)	(0.044)	(0.11)	(0.11)
Household		−0.125^***^	−0.002	−0.005	−0.124^***^	−0.126^***^
		(0.013)	(0.029)	(0.005)	(0.013)	(0.013)
Edu		0.021^***^	−0.005	0.001	0.021^***^	0.021^***^
		(0.003)	(0.005)	(0.001)	(0.003)	(0.003)
Mar		−0.035^***^	0.007	0.007	−0.035^***^	−0.034^***^
		(0.014)	(0.01)	(0.005)	(0.014)	(0.014)
Chronic		−0.196^***^	0.012^***^	0.02^***^	−0.189^***^	−0.193^***^
		(0.004)	(0.002)	(0.002)	(0.004)	(0.004)
Insurance		0.069^**^	0.044^***^	0.052^***^	−0.06^**^	−0.061^**^
		(0.028)	(0.014)	(0.007)	(0.027)	(0.028)
GDPP		−2.37e-07^***^	2.50E-07	2.17e-08^*^	−2.50e-07^***^	−2.34e-07^***^
		(3.51E-08)	(2.42E-07)	(1.18E-08)	(3.51E-08)	(3.50E-08)
Urban		0.0001^***^	−0.0001	−0.0001^*^	0.0001^***^	0.0001^***^
		(0.001)	(0.001)	(0.001)	(0.001)	(0.002)
OS					−0.182^***^	
					(0.012)	
IS						−0.155^**^
						(0.015)
Constant	3.041^***^	3.621^***^	0.234^**^	−0.122^***^	3.639^***^	3.603^***^
	(0.005)	(0.054)	(0.084)	(0.018)	(0.054)	(0.054)
City FE	YES	YES	YES	YES	YES	YES
Year FE	YES	YES	YES	YES	YES	YES
N	35,124	35,124	35,124	35,124	35,124	35,124
*R* ^2^	0.002	0.061	0.003	0.012	0.067	0.064

The symbol ^***^,

^**^,

* *indicates that the correlation coefficients are statistically significant at 1, 5, and 10%, respectively*.

H2a and H2b relate to the mediating effect of outpatient service availability and inpatient service availability on resident health, respectively. Based on Models (1) and (2), the results of Models (3) and (4) show that smart healthcare services are negatively correlated with outpatient service availability (*p* < 0.01), while smart healthcare service is positively correlated with inpatient service availability (*p* < 0.05). After adding the mediating variables, the coefficients of smart healthcare services in Models (5) and (6) were both lower than those in Model (2) and significant (*p* < 0.01). The results indicate that smart health services can improve resident health by decreasing the usage of outpatient services and enhancing the availability of inpatient services. Thus, H2a and H2b were supported.

### Robustness Test

Considering the possible reverse causality between smart healthcare services and resident health, we used the instrumental variable method to solve the endogeneity issue (51). We chose the number of fixed telephones in 1996 as the instrumental variable for smart healthcare services. From the relevant perspective of instrumental variables, the development of smart healthcare services mainly depends on the popularization of Internet technology. Areas with a higher penetration rate of fixed telephones are likely to be areas with a more complete digital medical infrastructure. From the exogenous perspective of the instrumental variable, the use frequency of fixed telephones is declining with the progress of new-generation information technology. The impact of fixed telephones on residents gradually disappears, which is consistent with the uniqueness of the instrumental variables. As the samples in this study were panel data, we added a variable that changed over time to construct an interaction term (52). Specifically, we used the number of fixed telephones per 100 people in each city in 1996 and the number of Internet broadband users in China to construct an interaction term as the instrumental variable of smart healthcare service that year.

Table 5 reports the 2SLS regression result of smart healthcare service and resident health. Considering endogeneity, smart healthcare service is still positively correlated with resident health. In Models (7) and (8), the Kleibergen-Paap rk's LM statistics *p*-value is <0.001, which significantly rejects the original hypothesis that identification of instrumental variables is insufficient. Kleibergen-Paap rk's Wald F statistic exceeds the critical value of the Stock-Yogo weak recognition test at the 10% level, which significantly rejects the hypothesis that identification of instrumental variables is weak. The results further verify the rationality and effectiveness of the instrumental variables in this study.

**Table 5:** The 2SLS regression result of robustness test.

	**(7)**	**(8)**
**Variable**	**Health**	**Health**
lv_SS	0.02^***^	0.014^***^
	−0.002	−0.002
Control Variables	NO	YES
City FE	YES	YES
Year FE	YES	YES
Kleibergen-Paap rk LM	910.58	1030.38
	[0.000]	[0.000]
Kleibergen-Paap rk Wald F	4685.3	4488.9
	{16.38}	{16.38}
N	35124	35124
*R* ^2^	0.005	0.062

The symbol ^***^ indicates that the correlation coefficients are statistically significant at 1% level.

## Further Discussion

Owing to the diversity of resources and development stages, the impacts of smart healthcare services on resident health may be heterogeneous in different regions. It is necessary to divide the sample into groups for further discussion. We selected economic regionalization and household registration types as grouping indicators. The former is divided into eastern, central, western, and northeastern regions according to the general practice of the existing literature. The latter is divided into urban and rural household registrations. Table 6 reports the descriptive statistics of the smart healthcare service and resident health for different economic regionalization and household registration types.

**Table 6:** The descriptive statistics of subgroup samples.

**Regional category**	**Name**	**N**	**Resident health**		**Smart healthcare services**	
			**Mean**	**SD**	**Mean**	**SD**
Economic regionalization	Eastern region	12,912	3.185	0.99	8.69E-10	1.412
	Central region	11,164	2.968	0.917	3.99E-09	1.342
	Western region	6,940	2.897	0.834	5.10E-09	1.488
	Northeast region	4,008	3.015	1.018	1.10E-08	1.095
Household	Urban	6,651	3.153	0.904	−2.02E-09	1.364
Registration types	Rural	28,473	3.014	0.957	−7.59E-10	1.344

Table 7 reports the regression results of the subgroup samples. As shown in Models (9–12), the impact of smart healthcare services on the resident health in the eastern region is significantly positive (*p* < 0.01), while in the central, western, and northeastern regions it is non-significantly negative (*p* > 0.1). On the one hand, as the Internet and digital infrastructure in the eastern region are more developed than other regions, the potentiality of smart healthcare service is fully released. On the other hand, the network diffusion effect of smart healthcare service is relatively low in these areas where smart infrastructure is not well-equipped. As a result, residents will obtain information and healthcare services at relatively higher cost, which is not conduce to health improvements.

**Table 7:** The regression results of the subgroup samples.

**Variable**	**(9)**	**(10)**	**(11)**	**(12)**	**(13)**	**(14)**
	**Eastern region**	**Central region**	**Western region**	**Northeast region**	**Urban**	**Rural**
SS	0.021^***^	−0.035	−0.005	−0.042	0.005	0.021^***^
	(0.008)	(0.009)	(0.04)	(0.067)	(0.008)	(0.004)
Control Variables	YES	YES	YES	YES	YES	YES
Constant	3.37^***^	3.628^***^	4.759^***^	3.382^***^	3.739^***^	3.489^***^
	(0.096)	(0.101)	(0.883)	(0.878)	(0.103)	(0.059)
City FE	YES	YES	YES	YES	YES	YES
Year FE	YES	YES	YES	YES	YES	YES
N	14,167	11,164	6,940	4,008	6,651	28,473
*R* ^2^	0.052	0.072	0.014	0.03	0.059	0.059

The symbol ^***^ indicates that the correlation coefficients are statistically significant at 1% level.

Models (13) and (14) show that the impact of smart healthcare services on rural resident health is significantly positive, while the impact on urban resident health is positive but not significant. This result can be explained in two ways. Restricted by economy, urbanization, and transportation, the healthcare conditions in rural areas lag far behind those in urban areas. The development of regional smart healthcare services, such as telemedicine, is conducive to overcoming the shortage and unequal distribution of healthcare resources in rural areas, which improves the service capacity of rural healthcare institutions (53). Owing to the limitations of network infrastructure and education, the proportion of urban residents using the Internet is remarkably higher than that of rural residents. Consequently, rural residents affected by the smart healthcare service have greater changes in the behavior of utilizing healthcare services, thereby improving their health more significantly.

## Conclusions

This study empirically examined the impact of regional smart healthcare services on resident health as well as the underlying mechanism by employing a two-way fixed effects model. The results showed that (1) smart healthcare services have a significant positive impact on resident health. (2) The availability of outpatient services and inpatient services plays a mediating role in the relationship between regional smart healthcare services and resident health. (3) The influence of regional smart healthcare services on resident health is heterogeneous among different regions. Specifically, the effect of smart healthcare services on resident health is significant in the eastern regions, while it is not significant in the central, western, and northeastern regions. The effect of smart healthcare services on resident health is significant in rural regions but not in urban regions.

### Theoretical Contributions

This study makes several theoretical contributions to the literature. First, this study enriches the research perspective of smart healthcare services. In the field of smart healthcare services, most contributions are focused on the drivers, barriers, system design, and implications of the smart healthcare service from the micro-organizational perspective, while this study clarified the impact and underlying mechanism of smart healthcare services that enable resident health from a macro-regional perspective. This study measured the regional smart healthcare service more comprehensively and reasonably by integrating data from three dimensions: regional, industrial, and organizational. Therefore, the paper fills the research gap in relation to the role of smart healthcare services in resident health at the regional level. Second, this article enriches the literature on the performance of smart healthcare services by empirically investigating the path through which smart healthcare services improve resident health. The existing studies mainly focused on examining the effects of smart healthcare services on health crisis management and maternal and neonatal care (54, 55). However, these studies failed to systematically analyze the impact path of smart healthcare services on resident health. This study attempts to clarify the mechanism by which smart healthcare services affect resident health by incorporating the two mediating factors from the perspective of healthcare service availability. Third, this paper expands research on dwindling the differences in various regions through smart healthcare services by analyzing the influence of smart healthcare services on resident health in different economic zones and urban and rural areas. Effectively reducing the disparity in healthcare among regions is essential for the sustainable development of society (56). This study verifies the proposition that the combination of traditional medicine and intelligent technologies has positive influence on narrowing the regional medical gap.

### Practical Implications

This study also offers some practical implications for policymakers. First, as revealed by research findings, the government should pay attention to increasing investment in smart healthcare service infrastructure as smart healthcare services are characterized by network externalities. This is instrumental in the rapid emergence of increasing marginal revenue of smart healthcare services and narrowing the digital gap in different regions, which will enhance the healthy life of residents. Second, the medical institutions should make attempts to embedding information technology into the traditional health mode for the realization of digital transformation. Medical institutions should improve the utilization and efficiency of outpatient and inpatient resources through digital technology, which makes for providing more efficient and high-quality medical services to resident. Third, in the context of the COVID-19 pandemic, it is necessary to utilize the advantages of telemedicine in reducing face-to-face contact and preventing the risk of cross-infection, which is helpful in improving the efficiency of epidemic prevention. Lastly, as confirmed of our study, more healthcare input should be implemented in rural areas as the development of regional smart healthcare services can contribute to overcoming the shortage and unequal distribution of healthcare resources in rural areas. The government should implement dynamic and differentiated smart service strategies to narrow the regional medical gap between urban and rural areas, thus achieving the strategic objective of Healthy China.

### Limitations and Future Research Directions

This study still has certain limitations, which represents the future research directions. First, due to the availability of data, the study fails to explore the role of smart services in COVID-19. This topic becomes more relevant since the COVID-19 has been the most serious public crisis since World War II and has posed a great challenge to the healthcare service system all over the world. Hence, once relevant data is available, future study could further verify whether the theoretical mechanism proposed by this paper is still effective in the context of serious public health emergencies. Second, the generalizability of this research findings is limited by the sample size, region and country sources Further research could collect data from other regions and countries to compare whether the effects of smart healthcare services are different between emerging economies and developed countries.

## Data Availability Statement

The original contributions presented in the study are included in the article/supplementary materials, further inquiries can be directed to the corresponding author.

## Author Contributions

YC: conceptualization, methodology, and writing original draft. LZ: software, formal analysis, and writing—review and editing. MW: investigation and data curation. All authors made contribution to the work and approved the submitted version.

## Funding

We acknowledge the financial support from the BUPT Excellent Ph.D. Students Foundation (Grant No. CX2019129) and the National Natural Science Foundation of China (Grant No. 7217020799).

## Conflict of Interest

The authors declare that the research was conducted in the absence of any commercial or financial relationships that could be construed as a potential conflict of interest.

## Publisher's Note

All claims expressed in this article are solely those of the authors and do not necessarily represent those of their affiliated organizations, or those of the publisher, the editors and the reviewers. Any product that may be evaluated in this article, or claim that may be made by its manufacturer, is not guaranteed or endorsed by the publisher.

## References

[1] Kumar P Singh SK Pereira V Leonidou E. Cause-related marketing and service innovation in emerging country healthcare. Int Mark Rev. (2020) 7:803–27. 10.1108/IMR-03-2019-0101

[2] Li L Chen Q Powers D. Chinese Healthcare Reform. Mod China. (2012) 38:630–45. 10.1177/0097700412457913

[3] Pelcastre-Villafuerte BE Meneses-Navarro S Ruelas-González MG Reyes-Morales H Amaya-Castellanos A Taboada A. Aging in rural, indigenous communities: an intercultural and participatory healthcare approach in Mexico. Ethn Health. (2017) 22:610–30. 10.1080/13557858.2016.1246417 27788597

[4] Lyng HB Ree E Wibe T Wiig S. Healthcare leaders' use of innovative solutions to ensure resilience in healthcare during the Covid-19 pandemic: a qualitative study in Norwegian nursing homes and home care services. BMC Health Serv Res. (2021) 21:878. 10.1186/s12913-021-06923-1 34446000 PMC8390181

[5] Tian S Yang W Grange JM. Le, Wang P, Huang W, Ye Z. Smart healthcare: making medical care more intelligent. Glob Heal J. (2019) 3:62–5. 10.1016/j.glohj.2019.07.001

[6] Liu K Tao D. The roles of trust, personalization, loss of privacy, and anthropomorphism in public acceptance of smart healthcare services. Comput Human Behav. (2022) 127:107026. 10.1016/j.chb.2021.107026

[7] Pan J Ding S Wu D Yang S Yang J. Exploring behavioural intentions toward smart healthcare services among medical practitioners: a technology transfer perspective. Int J Prod Res. (2019) 57:5801–20. 10.1080/00207543.2018.1550272

[8] Su Y Hou F Qi M Li W Ji Y. A data-enabled business model for a smart healthcare information service platform in the era of digital transformation. J Healthc Eng. (2021) 2021:5519891. 10.1155/2021/5519891 34158912 PMC8187060

[9] Sukkird V Shirahada K. Technology challenges to healthcare service innovation in aging Asia: Case of value co-creation in emergency medical support system. Technol Soc. (2015) 43:122–8. 10.1016/j.techsoc.2015.08.002

[10] Prati A Shan C Wang KIK. Sensors, vision and networks: from video surveillance to activity recognition and health monitoring. J Amb Intel Smart En. (2019) 11:5–22. 10.3233/AIS-180510

[11] Seifert R Weber M Kocakavuk E Rischpler C Kersting D. Artificial intelligence and machine learning in nuclear medicine: future perspectives. Semin Nucl Med. (2021) 51:170–7. 10.1053/j.semnuclmed.2020.08.003 33509373

[12] Alonso SG Arambarri J López-Coronado M. de la Torre Díez I. Proposing new blockchain challenges in ehealth. J Med Syst. (2019) 43:64. 10.1007/s10916-019-1195-7 30729329

[13] Chanson M Bogner A Bilgeri D Fleisch E Wortmann F. Blockchain for the IoT: privacy-preserving protection of sensor data. J Assoc Inf Syst. (2019) 20:1272–307. 10.17705/1jais.00567

[14] Hameed K Bajwa IS Sarwar N Anwar W Mushtaq Z Rashid T. Integration of 5G and block-chain technologies in smart telemedicine using IoT. J Healthc Eng. (2021) 22:1–18. 10.1155/2021/8814364 33824715 PMC8007349

[15] Parnell K Kuhlenschmidt K Madni D Chernyakhovsky C Donovan I Garofalo K. Using telemedicine on an acute care surgery service: improving clinic efficiency and access to care. Surg Endosc. (2021) 35:5760–5. 10.1007/s00464-020-08055-9 33048233

[16] Xu H Huang S Qiu C Liu S Deng J Jiao B. Monitoring and management of home-quarantined patients with COVID-19 using a wechat-based telemedicine system: retrospective cohort study. J Med Internet Res. (2020) 22:e19514. 10.2196/19514 32568727 PMC7333794

[17] Elayan H Aloqaily M Guizani M. Digital twin for intelligent context-aware IoT healthcare systems. IEEE Internet Things J. (2021) 8:16749–57. 10.1109/JIOT.2021.305115827295638

[18] Alhamid MF. Investigation of mammograms in the cloud for smart healthcare. Multimed Tools Appl. (2019) 78:8997–9009. 10.1007/s11042-017-5239-z

[19] de Redon E Centi A. Realities of conducting digital health research: challenges to consider. Digit Heal. (2019) 5:205520761986946. 10.1177/2055207619869466 31448129 PMC6689913

[20] Jin H-Y Liu X Xue Q-L Chen S Wu C. The association between frailty and healthcare expenditure among chinese older adults. J Am Med Dir Assoc. (2020) 21:780–5. 10.1016/j.jamda.2020.03.008 32331768

[21] Zhang C Lei X Strauss J Zhao Y. Health insurance and health care among the mid-aged and older Chinese: evidence from the national baseline survey of CHARLS. Health Econ. (2017) 26:431–49. 10.1002/hec.3322 26856894 PMC4980285

[22] Barbosa W Zhou K Waddell E Myers T Dorsey ER. Improving access to care: telemedicine across medical domains. Annu Rev Public Health. (2021) 42:463–81. 10.1146/annurev-publhealth-090519-093711 33798406

[23] Salman OH Aal-Nouman MI Taha ZK. Reducing waiting time for remote patients in telemedicine with considering treated patients in emergency department based on body sensors technologies and hybrid computational algorithms: toward scalable and efficient real time healthcare monitoring system. J Biomed Inform. (2020) 112:103592. 10.1016/j.jbi.2020.103592 33091572

[24] Alnoman A Anpalagan A. Towards the fulfillment of 5G network requirements: technologies and challenges. Telecommun Syst. (2017) 65:101–16. 10.1007/s11235-016-0216-9

[25] Pundziene A Heaton S Teece DJ. 5G, dynamic capabilities and business models innovation in healthcare industry. in 2019 IEEE International Symposium on Innovation and Entrepreneurship (TEMS-ISIE). Hangzhou: IEEE (2019). pp. 1–8.

[26] Nilashi M Ahmadi H Shahmoradi L Ibrahim O Akbari E. A predictive method for hepatitis disease diagnosis using ensembles of neuro-fuzzy technique. J Infect Public Health. (2019) 12:13–20. 10.1016/j.jiph.2018.09.009 30293875

[27] Miller AR Tucker C. Health information exchange, system size and information silos. J Health Econ. (2014) 33:28–42. 10.1016/j.jhealeco.2013.10.004 24246484

[28] McIntyre DP Chintakananda A. Competing in network markets: can the winner take all? Bus Horiz. (2014) 57:117–25. 10.1016/j.bushor.2013.09.005

[29] Chu H-L Chiang C-Y. The effects of strategic hospital alliances on hospital efficiency. Serv Ind J. (2013) 33:624–35. 10.1080/02642069.2011.622367

[30] Spanò R Di Paola N Bova M Barbarino A. Value co-creation in healthcare: evidence from innovative therapeutic alternatives for hereditary angioedema. BMC Health Serv Res. (2018) 18:571. 10.1186/s12913-018-3389-y 30029666 PMC6053759

[31] Shirazi F Wu Y Hajli A Zadeh AH Hajli N Lin X. Value co-creation in online healthcare communities. Technol Forecast Soc Change. (2021) 167:120665. 10.1016/j.techfore.2021.120665

[32] Farahani B Firouzi F Chang V Badaroglu M Constant N Mankodiya K. Towards fog-driven IoT eHealth: promises and challenges of IoT in medicine and healthcare. Futur Gener Comput Syst. (2018) 78:659–76. 10.1016/j.future.2017.04.036

[33] Shaban-Nejad A Michalowski M Buckeridge DL. Health intelligence: how artificial intelligence transforms population and personalized health. Npj Digit Med. (2018) 1:53. 10.1038/s41746-018-0058-9 31304332 PMC6550150

[34] Brown ME Bindman AB Lurie N. Monitoring the consequences of uninsurance: a review of methodologies. Med Care Res Rev. (1998) 55:177–210. 10.1177/107755879805500203 9615562

[35] Gunasekeran D V Ting DSW Tan GSW Wong TY. Artificial intelligence for diabetic retinopathy screening, prediction and management. Curr Opin Ophthalmol. (2020) 31:357–65. 10.1097/ICU.0000000000000693 32740069

[36] Safavi K Dare F. Virtual health care could save the U.S. billions each year. Harv Bus Rev. (2018).

[37] Rush KL Seaton C Li E Oelke ND Pesut B. Rural use of health service and telemedicine during COVID-19: the role of access and eHealth literacy. Health Informatics J. (2021) 27:146045822110200. 10.1177/14604582211020064 34041936

[38] Zhu H Guo Y Zhang L. An improved convolution Merkle tree-based blockchain electronic medical record secure storage scheme. J Inf Secur Appl. (2021) 61:102952. 10.1016/j.jisa.2021.102952

[39] Lu X Zhang R. Impact of physician-patient communication in online health communities on patient compliance: cross-sectional questionnaire study. J Med Internet Res. (2019) 21:e12891. 10.2196/12891 31094342 PMC6535977

[40] Nong S Chen Z. Whither the roads lead to? estimating association between urbanization and primary healthcare service use with chinese prefecture-level data in 2014. PLoS ONE. (2020) 15:e0234081. 10.1371/journal.pone.0234081 32492048 PMC7269333

[41] Xiao Y Qiu Q Huang Y Zhu S. Patients gather in large hospitals: the current situation of Chinese hospitals and the direction of medical reform. Postgrad Med J. (2021). 10.1136/postgradmedj-2021-140147. [Epub ahead of print]. 33879544

[42] Elleuch MA Hassena A. Ben, Abdelhedi M, Pinto FS. Real-time prediction of COVID-19 patients health situations using Artificial Neural Networks and Fuzzy Interval Mathematical modeling. Appl Soft Comput. (2021) 110:107643. 10.1016/j.asoc.2021.107643 34188610 PMC8225317

[43] Chen J See KC. Artificial intelligence for COVID-19: rapid review. J Med Internet Res. (2020) 22:e21476. 10.2196/21476 32946413 PMC7595751

[44] Xu B Li L Hu D Wu B Ye C Cai H. Healthcare data analysis system for regional medical union in smart city. J Manag Anal. (2018) 5:334–49. 10.1080/23270012.2018.149021134967848

[45] Gopi R Muthusamy P Suresh PG Gabriel Santhosh Kumar CV Pustokhina IA Pustokhin D. Optimal Confidential Mechanisms in Smart City Healthcare. Comput Mater Contin. (2022) 70:4883–96. 10.32604/cmc.2022.019442

[46] Kashef M Visvizi A Troisi O. Smart city as a smart service system: human-computer interaction and smart city surveillance systems. Comput Human Behav. (2021) 124:106923. 10.1016/j.chb.2021.10692326799652

[47] Maddox GL. Some correlates of differences in self-assessment of health status among the elderly. J Gerontol. (1962) 17:180–5. 10.1093/geronj/17.2.180 14468107

[48] Jylhä M. What is self-rated health and why does it predict mortality? Towards a unified conceptual model. Soc Sci Med. (2009) 69:307–16. 10.1016/j.socscimed.2009.05.013 19520474

[49] Solanas A Patsakis C Conti M Vlachos I Ramos V Falcone F. Smart health: a context-aware health paradigm within smart cities. IEEE Commun Mag. (2014) 52:74–81. 10.1109/MCOM.2014.687167327295638

[50] Dong E Liu S Chen M Wang H Chen L-W Xu T. Differences in regional distribution and inequality in health-resource allocation at hospital and primary health centre levels: a longitudinal study in Shanghai, China. BMJ Open. (2020) 10:e035635. 10.1136/bmjopen-2019-035635 32690509 PMC7371131

[51] Liu L. Emerging study on the transmission of the Novel Coronavirus (COVID-19) from urban perspective: evidence from China. Cities. (2020) 103:102759. 10.1016/j.cities.2020.102759 32501355 PMC7252103

[52] Nunn N Qian N. US Food aid and civil conflict. Am Econ Rev. (2014) 104:1630–66. 10.1257/aer.104.6.1630

[53] He C Zhou Q Chen W Tian J Zhou L Peng H. Using an internet-based hospital to address maldistribution of health care resources in rural areas of guangdong province, china: retrospective and descriptive study. JMIR Med Informatics. (2018) 6:e51. 10.2196/medinform.9495 30578195 PMC6320436

[54] Sondaal SFV Browne JL Amoakoh-Coleman M Borgstein A Miltenburg AS Verwijs M. Assessing the effect of mhealth interventions in improving maternal and neonatal care in low- and middle-income countries: a systematic review. PLoS ONE. (2016) 11:e0154664. 10.1371/journal.pone.0154664 27144393 PMC4856298

[55] Saeed T Kiong Loo C Shahreeza Safiruz Kassim M. Artificial intelligence based sentiment analysis for health crisis management in smart cities. Comput Mater Contin. (2022) 71:143–57. 10.32604/cmc.2022.021502

[56] Qiu Y Lu W Guo J Sun C Liu X. Examining the urban and rural healthcare progress in big cities of china: analysis of monitoring data in dalian from 2008 to 2017. Int J Environ Res Public Health. (2020) 17:1148. 10.3390/ijerph17041148 32059464 PMC7068349

